# A mixed-methods evaluation of interprofessional education in palliative care: changes in student attitudes towards health professions

**DOI:** 10.3205/zma001500

**Published:** 2021-09-15

**Authors:** Peggy Borchers, Martin Bortz, Henriette Hoffmann, Kristin Seele, Jeannine Schübel

**Affiliations:** 1 Technische Universität Dresden, Medizinische Fakultät, Bereich Allgemeinmedizin, Dresden, Germany; 2 Technische Universität Dresden, Medizinische Fakultät, Medizinisches Interprofessionelles Trainingszentrum, Dresden, Germany

**Keywords:** interprofessional education, teaching, palliative care, health professions

## Abstract

**Background: **Future health care increasingly requires interprofessional thinking and decision-making which should be taught during medical study and vocational training. Against this backdrop, the Medical Faculty at TU Dresden developed an elective course on “Interprofessional Palliative Medicine” in which medical students and trainees in different health professions have been taught together since the 2017 summer semester. An extensive and simultaneous course evaluation conducted in the 2019 summer semester and 2019/20 winter semester investigated if and how strongly attendees’ perceptions of interprofessional collaboration had changed as a result of the elective course.

**Method:** The course evaluations included quantitative pre- and post-questions on a questionnaire (n=50) covering, among other things, the perception of roles, according to the Role Perception Questionnaire, and qualitative interviews (n=20). The pre- and post-questions were compared using the Wilcoxon test for related samples and the effect sizes were calculated according to Cohen. The qualitative interviews were analyzed for content using a combined deductive-inductive approach.

**Results:** It was seen that the perceptions and attitudes of each professional group were mutually influenced as a result of the elective course. The quantitative analysis showed the largest effects regarding gains in understanding the roles and competencies of one’s own and the other professions (d=0.975) and a reciprocal feeling of “being dependent on each other” (d=0.845). In the interviews, it was seen that medical students developed a greater appreciation for the subject matter and tasks associated with nursing. A strengthening of self-perception was primarily found in the trainees.

**Conclusion: **The elective course on “Interprofessional Palliative Medicine” contributed to the changes in attitude not only with an increased self-awareness of one’s own professional group, but also a greater understanding of the roles and expertise of the other health professions. The results speak for the benefit of expanding the interprofessional courses offered.

## Zusammenfassung

**Hintergrund: **Die zukünftige Gesundheitsversorgung erfordert zunehmend interprofessionelles Denken und Handeln, welches bereits im Studium und in der Ausbildung vermittelt werden sollte. Vor diesem Hintergrund entwickelte die Medizinische Fakultät der TU Dresden das Wahlfach „Interprofessionelle Palliativmedizin“, in dem Medizinstudierende sowie Auszubildende verschiedener Gesundheitsfachberufen seit dem Sommersemester 2017 gemeinsam unterrichtet wurden. Eine umfangreiche begleitende Lehrevaluation im Sommersemester 2019 und Wintersemester 2019/2020 ging den Fragen nach, ob - und wie stark - sich durch das Wahlfach Wahrnehmungen der Teilnehmer in Bezug auf interprofessionelle Zusammenarbeit verändert haben.

**Methodik: **Die Lehrevaluation umfasste quantitative Prä- und Post-Befragungen per Fragebögen (n=50), unter anderem zur Rollenwahrnehmung nach dem Role Perception Questionnaire, sowie qualitative Interviews (n=20). Für Vergleiche zwischen der Prä- und Post-Befragung wurden der Wilcoxon Test für verbundene Stichproben durchgeführt sowie die Effektstärken nach Cohen berechnet. Die qualitativen Interviews wurden inhaltsanalytisch mit einen kombiniert deduktiv-induktiven Vorgehen ausgewertet.

**Ergebnisse: **Es zeigte sich, dass durch das Wahlfach die Wahrnehmungen und Einstellungen der jeweiligen Berufsgruppen wechselseitig beeinflusst wurden. Die quantitative Analyse zeigte die größten Effekte bezüglich eines Verständniszuwachses für die Rollen und Kompetenzen der eigenen (und anderer) Berufsgruppen (d=0,975) sowie eines gegenseitigen „Aufeinander-angewiesen-Seins“ (d=0,845). In den Interviews zeigte sich, dass Studierende der Medizin durch das Wahlfach eine höhere Wertschätzung für die Inhalte und Aufgaben der Pflege entwickeln konnten. Bei den Auszubildenden der Gesundheitsfachberufe wurde vor allem eine Stärkung der Selbstwahrnehmung verzeichnet.

**Schlussfolgerung: **Das Wahlfach „Interprofessionelle Palliativmedizin“ trägt zu Veränderung der Wahrnehmung mit besserer Selbstwahrnehmung der eigenen Berufsgruppe sowie größerem Verständnis für Rollen und Kenntnisse anderer Berufsgruppen bei. Die Ergebnisse sprechen für den Nutzen eines Ausbaus interprofessioneller Lehrangebote.

## 1. Background

Making teamwork more effective and expanding interprofessional collaboration are now considered to be key strategies to meet the challenges of patient care and the health care system [1]. Patients with complex medical issues rely on cooperation among many health care professions to ensure the highest quality of care possible [2]. So that practitioners are adequately prepared for this kind of collaboration, interprofessional subject matter and teaching formats should be integrated early in the curricula [3]. In addition to fostering understanding and respect for each professional role, there are indications that the quality of patient care could also be improved as a result [4], [5]. Against this background, new teaching strategies for interprofessional education and the adaption of both medical and health profession curricula have gained increasing importance [2], [6], [7], [8] in an educational and training system that is more monoprofessionally structured in its teaching and training of physicians and practitioners of other health professions [9], [10]. In particular, the academic debate about interprofessional education and work has intensified in recent years [11]. Even policymakers in 2017 stipulated that interprofessional education in the form of joint courses be included in the “Master Plan Medical Studies 2020” [12].

It is precisely in the context of palliative care, the care of the most severely ill and dying patients, that interprofessional collaboration is an essential and integral element [13]. According to the definition of the WHO, quality of life is improved by palliative care “through the prevention and relief of suffering” by treating “pain and other problems, physical, psychosocial and spiritual” [14]. This means that in addition to physicians and nursing staff, spiritual advisors, physiotherapists and other professionals also play a role. In this area of health care, the special needs of the affected patients require caregivers to have not only the necessary medical expertise, but also special skills and attitudes, regardless of their occupation [15], [16]. The professional groups who care for palliative patients must be equal partners in order to do justice to the different needs and wants of patients who are at the end of life [17]. A crucial requirement for this type of teamwork is knowledge on the part of the different caregivers about the skills and responsibilities of the others [18]. For these reasons, in 2017 the Department of General Practice at the Medical School of the Technische Universität Dresden, in cooperation with the Medizinische Interprofessionelle Trainingszentrum Dresden (MITZ) and the vocational school of the Carus Akademie at the Carl Gustav Carus University Hospital in Dresden, developed an elective course in palliative medicine as a model project in which medical students and trainees in other health professions are taught together.

### 1.1. Interprofessional education (IPE)

The aim of interprofessional education is to initiate productive collaboration early on between different professional groups to improve patient care [2]. Mutual understanding between the different professions plays a critical role in this [19]. Necessary core competencies for interprofessional collaborative practice have been identified by the Interprofessional Education Collaborative:

1. Mutual respect and appreciation for individuals of other professions,
2. knowledge of the roles, competencies and responsibilities of one’s own and those of other professions,
3. responsible interprofessional communication,
4. responsible planning, delivery and evaluation of the interprofessional collaborative practice [20].

To initiate interprofessional collaborative practice early on, it is necessary to take these competencies into consideration during university study and vocational training in the form of interprofessional teaching and learning. For this reason, these core competencies are also defined as learning objectives in the National Competency-based Catalogue of Learning Objectives for Undergraduate Medical Education (see chapter 8).

Even though several interprofessional courses already exist, it still often remains unclear how the perceptions of members of the different professional groups change and if the interprofessional education has an effect on a potentially improved interprofessional collaborative practice in the future [3], [21]. To fill in these blanks, the elective course described in the following was extensively evaluated with a specific focus on changes with regard to the core competencies of interprofessional collaborative practice.

#### 1.2. The elective course on interprofessional palliative medicine

Since 2017 at the Medical Faculty of Dresden, medical students in the clinical phase of study and trainees in different health professions (nursing, geriatric care, physiotherapy, etc.) have been taught in small groups as part of the required elective course in Interprofessional Palliative Medicine (IPPM). This elective entails 24 teaching units along with small-group seminars, observations, practical simulations and communication trainings. The course content centers on the treatment and care of common main symptoms and the communicative and psychosocial aspects of treatment. The learning objectives are based on the university curriculum put forth by the German Association for Palliative Care (communication, teamwork, cooperation with other professions). A central objective of the elective course is to raise attendees’ awareness of the importance of interprofessional collaboration in terms of their own future professional practice and, with this in mind, to develop a professional attitude. Just as much value was placed on interprofessionalism when choosing the course instructors in order to provide insights into different subject areas (medicine, nursing, psychology, occupational and music therapy, spiritual counseling, law/medical legislation). The elective course was designed for 24 attendees (12 medical students, 12 trainees in health professions).

#### 1.3. Aim

The aim of this paper is to present the quantitative and qualitative results of the course evaluation from the model project. Answers were sought to the following research questions:

1. Do perceptions and attitudes change in regard to the core competencies of interprofessional collaborative practice as a result of the elective course (and if they do, how strongly)?
2. Are there differences between the medical students and the vocational trainees?
3. Which changes in perception and attitude have the attendees specifically noticed?

## 2. Methods

### 2.1. Evaluation setting

The IPPM course evaluation that is considered in this paper was conducted in the 2019 summer semester and the 2019/20 winter semester. The content and delivery of the elective course were the same in both semesters and are thus comparable. The evaluation was done on the first two levels of Kirkpatrick’s four-level model [22]. Level 1, the reaction level, served to capture the satisfaction of the attendees (general evaluation of the course) and level 2 (learning) was used to determine attendees’ changes in perceptions and attitudes.

#### 2.2. Evaluation design

The evaluation followed a mixed-methods approach with a parallel design. This entailed simultaneous collection of quantitative and qualitative data with the goal of using both sets of data to gain a deeper and better understanding of the situation [23]. To accomplish this, identical quantitative pre- and post-questionnaires were used in both semesters, and qualitative interviews were held with the attendees following a uniform guideline. The interviews served to make the changes measured on the questionnaires more visible and better understood in their actual form.

#### 2.3. Data collection

##### a) Quantitative questionnaires

In order to quantitatively measure changes, a pre-questionnaire was used at the beginning of the course (first day of the seminar) and a post-questionnaire with identical items was used at the end of the course (last day of the seminar) (see attachment 1 ). The questionnaire was based on an abridged Role Perception Questionnaire (RPQ)) [24]. Seventeen items were chosen and can be found in the attachment 1 . A personal code was assigned to match pre- and post-data. Sociodemographic information (age, sex, educational level) was collected for comparison between the professional groups. Apart from these items, the questionnaire also contained items that are not relevant to the topic of this paper and are not included. The questionnaires were filled out by the attendees using pencil and paper at the end of the seminar session. Participation was voluntary.

##### b) Qualitative interviews

The interviews involved semi-standardized guided interviews [25]. The guideline was created as a team based on Helfferich’s SPSS principle [26]. Four open questions on the main categories and relevant structure-giving sub-questions were formulated. The interviews took place on the final day of the seminar. Participation was voluntary and all of the attendees had been invited to do so by the course coordinator. Four interviews were held simultaneously in an effort to limit the time required of the participants on the last day as much as possible. The interviews were held by staff members from the Department of General Practice who had not been involved in the elective course but had received internal interviewer training and had been instructed on following the guideline. The interviews were recorded on tape and then transcribed verbatim.

#### 2.4. Evaluation/data analysis

##### a) Quantitative data collection using questionnaires

The analysis of the questionnaires was carried out using the statistics software SPSS 25.0. The statistically significant level α was defined as 5% in all analyses. Non-parametric tests were applied due to the absence of a normal distribution of the data.

To answer the **first research** **question**, if and how strongly the IPPM elective course changes perceptions and attitudes toward interprofessional collaboration, comparisons were made between the pre- and post-questionnaires within a group. The differences in mean values (MV) were analyzed with the Wilcoxon test for related samples (exact test). The effect size was calculated according to Cohen to evaluate the strength and thus the meaningfulness of the results (in respect to a change in attitude). In the analysis of the effect size, the values of d=0.5 represent a medium effect size and d=0.8 a large one [27].

To answer the **second research question**, if differences exist between the medical students and the trainees in health professions, the mean values were compared between the two groups at the separate time points using the Mann-Whitney U test (exact test) for independent samples.

##### b) Qualitative interviews

To answer the **third research question**, which probes more deeply into the perceptions and attitudes, the interviews were analyzed using qualitative content analysis that was supported by the software MAXQDA. The qualitative content analysis was methodically carried out according to the content-structured approach of Philipp Mayring [28] based on a deductive system of categories. The deductive main categories were defined using the core competencies of the Interprofessional Education Collaborative (see table 1 (Tab. 1), main categories 1-4). The subcategories and the 5^th^ main category (benefit from topics pertaining to the other professions) were determined using an inductive approach [29] since some of the meanings only became visible upon examination of the texts (see table 1 (Tab. 1)). The coding was done independently by two research assistants (PB, MB) using the previously defined deductive coding scheme. All of the categories were discussed and adjusted in the process of performing the analysis. Furthermore, statements were identified to serve as anchor examples. A trial coding exercise took place based on the trial interview.

## 3. Results

### 3.1. Composition of the course 

In the 2019 summer semester, 23 people attended the IPPM elective course and 27 in the 2019/20 winter semester. Fifty percent of the 50 attendees were medical students (see table 2 (Tab. 2)). The trainees hailed from nursing (38%), geriatric care (10%) and pediatric nursing (2%). Table 2 (Tab. 2) gives an overview of the other sociodemographic characteristics of the attendees.

#### 3.2. Participation in the evaluation

The pre-questionnaire was filled out by all 50 attendees (response rate: 100%). Due to dropping the course or other reason for absence, a total of 41 attendees completed the post-questionnaire during the final session (response rate: 82%). Seven of the nine attendees who were absent were nursing trainees. The reasons for this remained unknown.

Twenty of the 50 attendees participated in the interviews (13 medical students, 7 trainees). The interviews lasted between 15 and 40 minutes.

#### 3.3. Changes in perceptions and attitudes

Significant differences were found for several questionnaire items between the pre- and post-questionnaires. A selection of relevant items and the calculated test results are presented in table 3 (Tab. 3). For a better overview, the items have been have been organized according to the four core competencies of the Interprofessional Education Collaborative model.

##### Mutual respect and appreciation for other professions

The trainees’ agreement with the statement, “Members of other professions respect the work done by members of my profession”, significantly improved at the end of the elective course (post) with a medium effect size (MV=2.2, d=0.632). Likewise, over the course of the elective the perceptions of all attendees (total) changed for the positive in reference to the “good relationships with members of other professions” (p=0-028).

##### Knowledge of the roles, competencies and responsibilities of one’s own and the other professions

After completing the elective course, all of the attendees were significantly better able to define the competencies/responsibilities and limits of their own (d=0.707) and the other professions (d=0.736). The largest effect was seen in the trainees’ definition of one’s own professional competencies/responsibilities and limits (d=0.975).

##### Interprofessional communication

The communication skills of the members of one’s own profession with other team members and patients was rated higher overall (MV differences: 0.21; 0.23) and in the separate groups at course end, but was insignificant.

##### Interprofessional teamwork

At the end of the course, all of the attendees gave a more positive rating to the statement that their professions were able to work well with others and that they are dependent upon the work done by other professions (from MV=1.69 to MV=1.32; d=0.508). The latter showed itself to be particularly pronounced in the trainees, with d=0.845.

#### 3.4. Differences between medical students and vocational trainees

Differences between the groups were seen for several items at the separate time points.

##### Mutual respect and appreciation for the other professions

It was found that at the beginning (pre) the trainees rated the respect that the other professions showed for their own significantly lower (MV=2.78) than the medical students (MV=1.88, p=0.000). At the end of the elective course, this difference between the groups was clearly smaller and no longer significant.

##### Knowledge of the roles, competencies and responsibilities of one’s own and the other professions

At course begin there were significant differences between the groups regarding the statement, “Members of my profession have an understanding of the knowledge, skills, roles and responsibilities of other professions” (p=0.001). These differences no longer existed at the end of the course (p=0.058). For this matter, the medical students’ understanding had become stronger by the time of the post-questionnaire.

##### Interprofessional communication

In regard to communication with patients and team members, significant differences between the groups were measured at both time points. At both time points the medical students rated their communication skills more poorly than the trainees did. However, the communication skills were rated higher by both groups at the end of the course.

##### Interprofessional teamwork

At the beginning of the elective course the perception of the trainees that their profession was dependent on other professions was less pronounced (MV=2.08) than the perception of medical students (MV=1.29). At course end both groups had moved closer together.

#### 3.5. Specific changes in the perceptions and attitudes of the attendees

In general, the interviews showed that the perception and understanding of the other professions had often not changed fundamentally, but rather had strengthened accordingly. The changes in perceptions and attitudes specifically noticed by the attendees are reflected in the subcategories with anchor examples in table 1 (Tab. 1).

##### Mutual respect and appreciation for the other professions

The interviewees reported a self-perceived increase in appreciation and a better understanding of other professions. This increased appreciation was mainly emphasized by the medical students, as illustrated by an excerpt from a statement in table 1 (Tab. 1): *“because it also includes an appreciation for all the participants and the meaning is simple: it doesn’t matter what you are studying or training to be, you are simply part of the same...” (T15, f, med.)*

##### Knowledge of the roles, competencies and responsibilities of one’s own and the other professions

Both groups perceived and reflected on the existing hierarchies between and also within the professions, as well as on the different kinds of patient interactions. One trainee (T1) articulated this concretely: “[…] *because it has very much been drummed into you or it is seen in daily practice that the physicians are way up top, the nurses way down below, and as trainee even lower”* (see table 1 (Tab. 1)).

##### Interprofessional communication

Many interviewees emphasized the importance of cooperative decision-making and communication between the different professions, and that communication at eye level is indeed possible. They recognized that sharing important information from another profession could contribute to joint decision-making and the inclusion and acceptance of another perspective can be made possible. This is clearly stated by a medical student (T7): “[…] *during this [interprofessional] team meeting you noticed how important the work is, and I believe in the advantages that can be gained from it when you sometimes just include another point of view”* (see table 1 (Tab. 1)).

##### Interprofessional teamwork

The interviewees discovered that everyone must have a feel for or knowledge of what “the other professions do” in order for interprofessional collaborative practice to be successful. The trainees primarily raised the issue of the currently existing, often historically based inhibitions in communicating with the physician. As a result of the close collaboration with the medical students, they have gathered courage in that they can and should speak with physicians as part of interprofessional collaborative practice.

##### Benefit from topics pertaining to the other professions

Furthermore, it was made very clear during the interviews that the otherwise unusual grappling with topics pertaining to the other professions (nursing, medical or therapeutic) was often perceived as a benefit. The majority of statements were made by medical students during the interviews in which they claimed that they felt they profited from looking more closely at nursing topics (patient positioning and aroma therapy mostly). Moreover, the interviewees often discovered that, at the same time, insight into the topics of other professions represented a benefit for the patients and patient care (for example, the topic of positioning patients), which is why this aspect was included as an additional main category.

## 4. Summary and discussion

The results of the evaluation show that the perceptions and attitudes of each professional group can be influenced and thus changed as a result of the interprofessional course (research question 1). The understanding of the benefits of interprofessional collaboration as an element of palliative care can be improved by teaching the different professions together. This can lay the foundation for effective future teamwork which is crucial to the care of the most severely ill and dying.

The largest effect sizes were seen regarding a gain in understanding the roles and responsibilities of one’s own profession (and the others) and a feeling of “being mutually dependent on each other.” These observations correspond with the results of other studies [4], [30]. It can be seen in the interviews that the willingness to engage in interprofessional dialogue can be a pre-requisite for changes in perceptions and attitudes. That this can be highly valued within the student body is seen in other studies [31]. Generally, it can be assumed that interprofessional education should be integrated as early as possible in the curriculum in order to foster these effects on perception and attitude [32].

In regard to the second research question, it was possible to show that differences in perception exist between the medical students and the trainees, but that these differences had become definitively smaller by the end of the elective course. There was, as a consequence, a certain amount of aligning or converging of specific attitudes. This particularly involved an increased awareness of the different understandings of the roles and knowledge of the responsibilities associated with the roles, which is an important basis for teamwork [18], [31]. This development represents an essential pre-requisite for working together as equal partners, an important requirement for interprofessional teams, as described above in the context of palliative care [17].

The parallel evaluation design enabled a deeper investigation of the changes, making it possible in the interviews to discover specific aspects through the lens of the attendees. In addition, it was evident that the attendees perceived the existence of different situations (see table 1 (Tab. 1): Perception of existing hierarchies) and their need for change.

It became clear that the groups were able to derive different benefits from the elective course. Trainees in the various health professions were particularly able to profit from the interprofessional education through an enhancement of their self-confidence and their communication skills. These results are consistent with those published in other studies [33] and underscore the value of communication modules in interprofessional education [33]. The initial differences can probably be explained by the traditional profession-specific socialization of the groups and their social identities that include internal professional cultures and status, their own values and standards [34], [35].

That certain topics were more frequently emphasized by medical students in the interviews (for instance, an increased appreciation of the other professions) can very likely be accounted for by the fact that more medical students participated in the interviews. However, it could also be an indication that the medical students had only first come into intense contact with other professions during the elective course and that they had reflected upon this experience. These results, in turn, suggest that interprofessional cooperation in the student body does indeed face barriers and challenges and overcoming them can be practiced in the educational setting [32]. Finally, medical students appear to benefit particularly from the content-based teaching of nursing skills.

In summary, this paper has helped identify how interprofessional education influences the perceptions of other professions.

## 5. Limitations

This evaluation study included two different semesters in order to have a sample size large enough to draw conclusions from. Comparability is possible due to the identical nature of the course in each semester. Nonetheless, the sample size of n=50 (questionnaires) and n=20 (interviews) must be taken into consideration when interpreting the results.

Attendance of the elective course and participation in the interviews were voluntary, which encourages selection bias. It can be assumed that prior to the course a greater appreciation and acceptance of interprofessional teamwork prevailed among the attendees, which is why (particularly in the interviews) only minor changes in attitude came to light. In addition, this can offer an explanation for the high response rate and the great willingness to participate in the interviews.

Furthermore, answers motivated by social desirability, a recall bias, or an interviewer bias cannot be ruled out.

This study is not in a position to show if the changes in attitude are sustained or if IPE also leads to actually improved interprofessional collaboration in the future.

## 6. Conclusions for teaching and practice

The required elective course on “Interprofessional Palliative Medicine” contributed to changes in perception, a greater understanding of the roles and knowledge of other professional groups, and thus appears to be suitable and important for early initiation of good interprofessional collaborative practice, which is important beyond the context of palliative care and is needed. Trainees in the different health professions benefit in particular from an appreciation of their roles and an improvement in their communication skills. Medical students profit from the teaching of nursing skills and an increased appreciation for the contributions of other professions in caring for patients. 

The results speak for and highlight the need to develop interprofessional education courses in order to meet the necessary and targeted goals of interprofessional collaborative practice and thus contribute in the long term to a high quality of patient care.

## Acknowledgements

The elective course was designed as a model project by the Department of General Practice at the Medical School in Dresden, in cooperation with MITZ and the Carus Akademie Dresden. We wish to thank the following people by name: Marie-Christin Willemer, Michael Sommer and Mario Zado. Special thanks also goes to all of the instructors and attendees who participated in this evaluation.

## Competing interests

The authors declare that they have no competing interests. 

## Supplementary Material

Questions on the pre- and post-questionnaire

## Figures and Tables

**Table 1:**
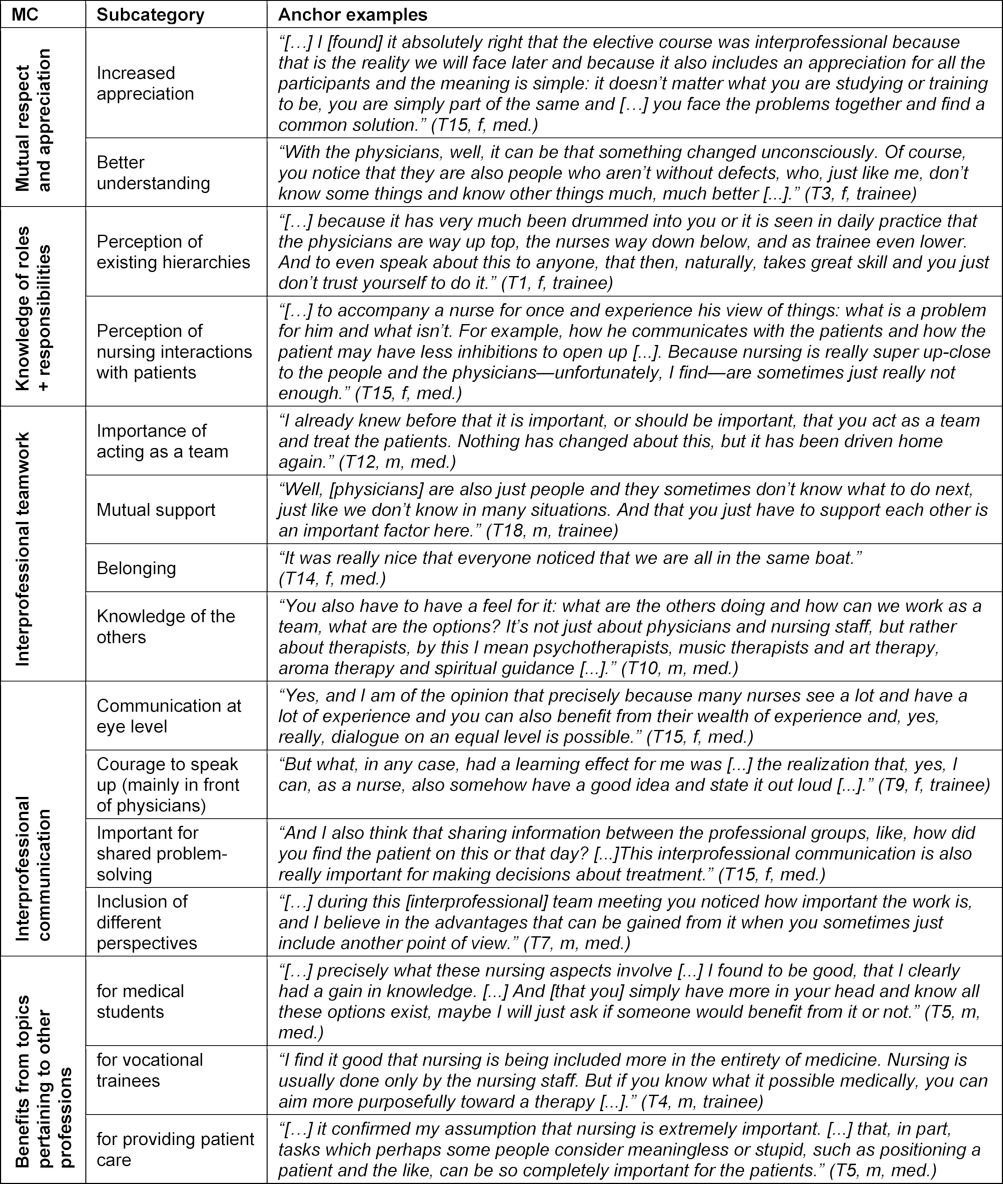
Important learning experiences and attitudes of the attendees taken from the interviews in regard to interprofessional collaborative practice (MC=main categories)

**Table 2:**
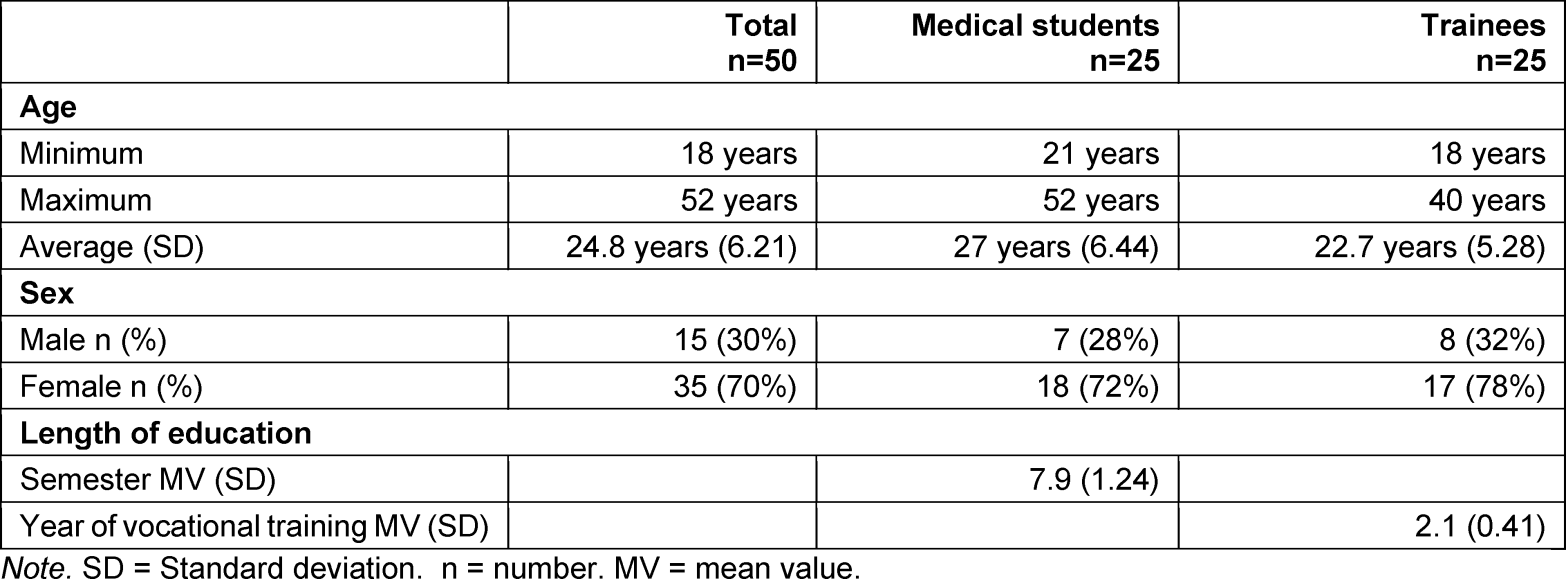
Sociodemographic characteristics of the attendees in both semesters

**Table 3:**
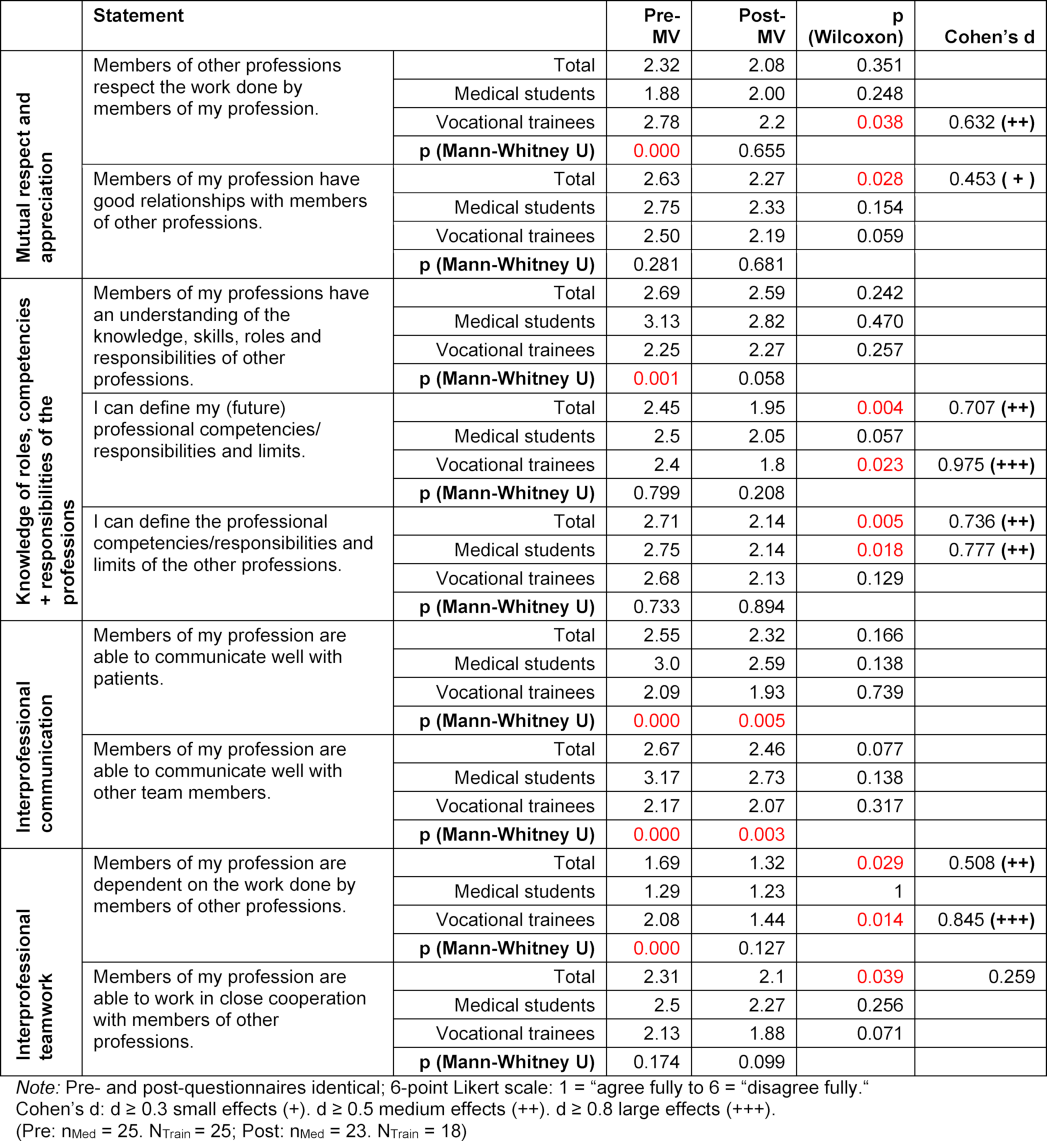
Evaluation of individual statements about one’s own or the other professions
