# Supplementary material for: A mixed-methods evaluation of interprofessional education in palliative care: changes in student attitudes towards health professions
Source: GMS J Med Educ. 2021 Sep 15;38(6):Doc104. doi: 10.3205/zma001500 (PMC8493842; doi:10.3205/zma001500)
Supplement: Questions on the pre- and post-questionnaire [file JME-38-6-104-s-001.pdf]

Attachment 1: **Questions on the pre- and post-questionnaire**

	Agree fully	Agree	Mostly agree	Mostly disagree	Disagree	Disagree fully
**Members of my profession:**						
are well trained.	O	O	O	O	O	O
are dependent on the work done by members of other professions.	O	O	O	O	O	O
are able to work in close cooperation with members of other professions.	O	O	O	O	O	O
are willing to share information and resources with members of other professions.	O	O	O	O	O	O
think highly of other professions with whom they have a relationship.	O	O	O	O	O	O
have good relationships with members of other professions.	O	O	O	O	O	O
have a higher status than members of other professions.	O	O	O	O	O	O
are scared of failing in front of others.	O	O	O	O	O	O
are able to communicate well with patients.	O	O	O	O	O	O
are able to communicate well with other team members.	O	O	O	O	O	O
have an understanding of the knowledge, skills, roles and responsibilities of other professions.	O	O	O	O	O	O
want to keep a professional distance.	O	O	O	O	O	O
have a lot of theoretical knowledge.	O	O	O	O	O	O
have a lot of practical knowledge/skills.	O	O	O	O	O	O
**Members of other professions** respect the work that is done by my profession.	O	O	O	O	O	O
**I can:**						
define my (future) professional competencies/responsibilities and limits.	O	O	O	O	O	O
define the professional competencies/responsibilities and limits of the other professions.	O	O	O	O	O	O

Attachment 1 to: Borchers P, Bortz M, Hoffmann H, Seele K, Schübel J. *A mixed-methods evaluation of interprofessional education in palliative care: changes in student attitudes towards health professions.* GMS J Med Educ. 2021;38(6):Doc104. DOI: 10.3205/zma001500
